# Traceable Reference Full Metrology Chain for Innovative Aspheric and Freeform Optical Surfaces Accurate at the Nanometer Level

**DOI:** 10.3390/s21041103

**Published:** 2021-02-05

**Authors:** Yassir Arezki, Rong Su, Ville Heikkinen, François Leprete, Pavel Posta, Youichi Bitou, Christian Schober, Charyar Mehdi-Souzani, Bandar Abdulrahman Mohammed Alzahrani, Xiangchao Zhang, Yohan Kondo, Christof Pruss, Vit Ledl, Nabil Anwer, Mohamed Lamjed Bouazizi, Richard Leach, Hichem Nouira

**Affiliations:** 1 Laboratoire Commun de Métrologie (LCM), Laboratoire National de Métrologie et d’Essais (LNE), 1 Rue Gaston Boissier, 75015 Paris, France; yassir.arezki@lne.fr; 2 Université Paris-Saclay, Université Sorbonne Paris Nord, ENS Paris-Saclay, LURPA, 91190 Gif-sur-Yvette, France; nabil.anwer@ens-paris-saclay.fr; 3 Manufacturing Metrology Team, Faculty of Engineering, University of Nottingham (UNOTT), Nottingham NG8 1BB, UK; rong.su@nottingham.ac.uk (R.S.); richard.leach@nottingham.ac.uk (R.L.); 4 VTT Technical Research Centre of Finland Ltd., Centre for Metrology MIKES, Tekniikantie 1, 02150 Espoo, Finland; ville.heikkinen@vtt.fi; 5 THALES LAS France, Etablissement de Saint Heand, Boulevard Ravel de Malval, 42570 Saint Heand, France; francois.lepretre@fr.thalesgroup.com; 6 TOPTEC, Institute of Plasma Physics of the Czech Academy of Sciences, Za Slovankou 1782/3, 182 00 Praha, Czech Republic; psota@ipp.cas.cz (P.P.); ledl@ipp.cas.cz (V.L.); 7 National Institute of Advanced Industrial Science and Technology (AIST), National Metrology Institute of Japan (NMIJ), Tsukuba, Ibaraki 305-8563, Japan; y-bitou@aist.go.jp (Y.B.); kondou.y@aist.go.jp (Y.K.); 8 Institute of Applied Optics (ITO), University Stuttgart, Pfaffenwaldring 9, 70569 Stuttgart, Germany; schober@ito.uni-stuttgart.de (C.S.); pruss@ito.uni-stuttgart.de (C.P.); 9 Department of Mechanical Engineering, College of Engineering, Prince Sattam bin Abdulaziz University (PSAU), Alkharj 16273, Saudi Arabia; ba.alzahrani@psau.edu.sa (B.A.M.A.); my.bouazizi@psau.edu.sa (M.L.B.); 10 Shanghai Engineering Centre of Ultra-Precision Optical Manufacturing, Fudan University, Shanghai 200438, China; zxchao@fudan.edu.cn

**Keywords:** robust reference minimum zone (Hybrid Trust Region) fitting, aspheric and freeform optical elements, ultra-high precision measuring machine, dimensional metrology, measured data evaluation, uncertainty

## Abstract

The design of innovative reference aspheric and freeform optical elements was investigated with the aim of calibration and verification of ultra-high accurate measurement systems. The verification is dedicated to form error analysis of aspherical and freeform optical surfaces based on minimum zone fitting. Two thermo-invariant material measures were designed, manufactured using a magnetorheological finishing process and selected for the evaluation of a number of ultra-high-precision measurement machines. All collected data sets were analysed using the implemented robust reference minimum zone (Hybrid Trust Region) fitting algorithm to extract the values of form error. Agreement among the results of several partners was observed, which demonstrates the establishment of a traceable reference full metrology chain for aspherical and freeform optical surfaces with small amplitudes.

## 1. Introduction

**Figure 1:**
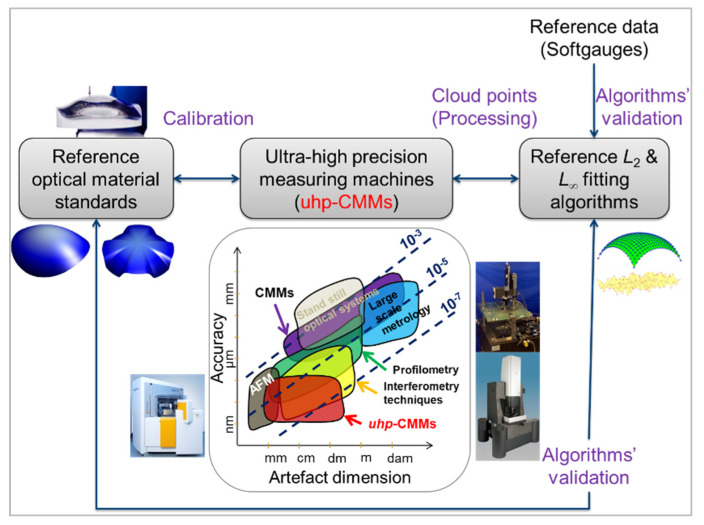
Description of the traceable reference full metrology chain for aspherical and freeform optical surfaces with respect to the SI unit meter definition. The traceable full metrology chain includes: high quality optical surfaces (1), ultra-high precision measurement machines (2) and robust reference algorithms (3) ensuring calculation error below the nanometer level.

With respect to the increasing demand for high quality aspherical and freeform optics in different fields (lithography, lasers, imaging, etc.) [1,2,3,4], metrology capabilities of aspheric and freeform surfaces must be aligned with advances made in the optical design domain [5]. The need for an accurate full metrology chain for asphere and freeform optics is not exclusively reserved to the extreme ultraviolet lithography (EUVL) [6], but it concerns also the synchrotron [7,8], astronomy [9,10], medical device [11], security and several other domains. The full metrology chain could include the development of robust reference mathematical least-squares (LS) and minimum zone (MZ) fitting algorithms, thermo-invariant material measures and ultra-high precision measuring machines (Figure 1); the three components are necessary for building the traceable full metrology chain at NMIs (national metrology institutes) and DIs (Designated Institutes).

Form error is a function of form deviations that define the orthogonal distances between the measured data points and the reference surface. When this function is taken as the difference between maximum and minimum deviations (PV: peak-to-valley), minimum zone is the least value of form error among all choices of reference surfaces [12]. Although there exist different methods to determine the so-called reference surface, no specific method is consensual. Among those methods, two approaches are extensively used in dimensional metrology, namely, least squares (LS) (also called Gaussian or L_2_ fitting) and minimum zone (MZ) (Chebyshev, L∞ fitting). The choice of approach depends on the parameters required. Thus, when the RMS (Root Mean Squares) is sought [13], LS fitting is preferred. In the case where the least value of PV is required, L∞ fitting is more suitable. Minimum zone is of crucial importance in form metrology. It indicates the form quality of the manufactured components. Nevertheless, for canonic surfaces such as cylindrical surface, a more clear definition still exists. Then, roundness could be estimated over the range 2–15 upr (undulation per revolution), while the waviness is assessed for the range 16–50 upr as indicated in [14,15,16]. Similar definition does not exist yet in the ISO standards for aspherical and freeform surfaces, which could represent an evident lack.

Form errors of aspheric and freeform surfaces have been traditionally estimated using LS fitting algorithms [12,17]. However, it has been shown that, in some circumstances, the LS method overestimates form error and hence can result in the rejection of conforming parts [18,19]. Recently, the MZ criterion has become popular since it conforms to the ISO Geometrical and Product Specifications [20]. MZ is more mathematically challenging than LS, especially for aspheric and freeform surfaces. Only a small number of MZ fitting algorithms, in particular Exponential Penalty Function (EPF) and Primal-Dual Interior Point Method (PDIP), for aspheric and freeform surfaces have been developed, implemented and validated using a number of reference softgauges [18].

A material measure is a realisation of the definition of a given quantity with a stated value and an associated measurement uncertainty [21]. Material measures allow the determination of the metrological characteristics of the instrument being calibrated [22,23], and only few have been developed using a high-precision process [5,24,25]. Additional innovative thermo-invariant material measures (TIMMs) have been designed in this work and manufactured using the original magnetorheological finishing (MRF) process. Two of these thermo-invariant material measures were used for the evaluation of a selected number of improved ultra-high precision measurement machines at the LNE (France), UNOTT (United Kingdom), IPP (Czech Republic), THALES-Agx (France), VTT (Finland), NMIJ (Japan) and ITO (Germany).

**Figure 2:**
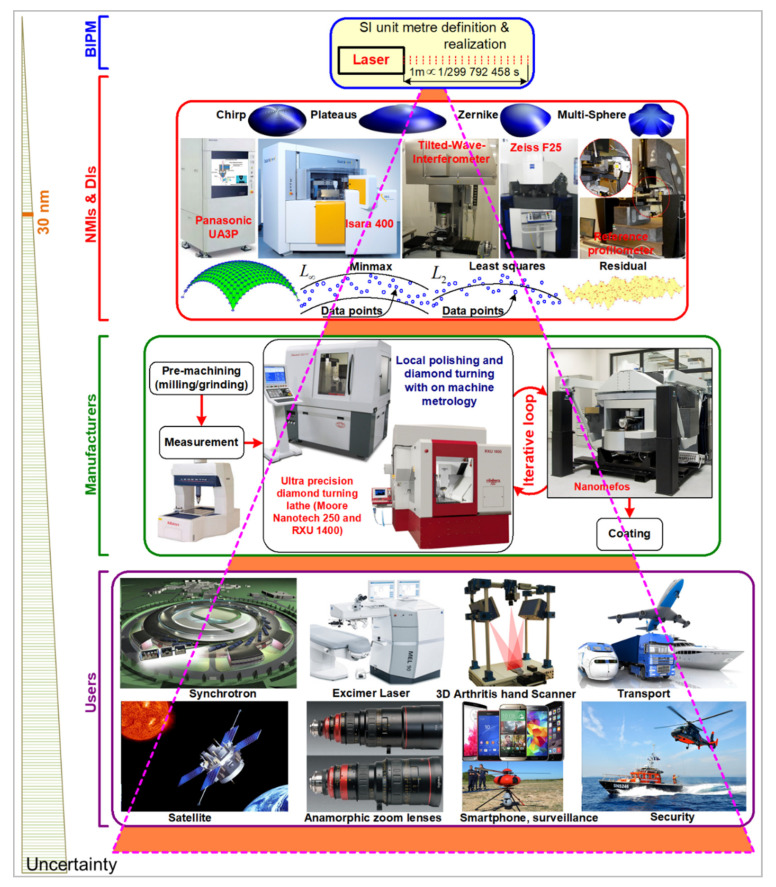
Description of the traceability chain in asphere and freeform metrology. The SI unit meter definition is established at the BIPM (International Bureau of Weights and Measures) by experts from NMIs (National Metrology Institutes) and DIs (Designated Institutes), materialised at NMIs and DIs and shared to manufactures and end users with respect to a pyramid organization.

Most of the existing ultra-precision reference single point instruments that could be used for the calibration of aspheric and freeform surfaces are briefly described in [23,26,27,28,29]. They are equipped with accurate optical and/or tactile probing systems, or optical imaging instruments such as the Tilted-Wave Interferometer (TWI) [30]. They could be used for the achievement of surface topography measurements with low uncertainties at the nanometer level in order to guarantee the best dissemination and transfer of the established traceable reference full metrology chain at NMIs and Dis to accredited laboratory and industry (Figure 2). Furthermore, modelling the physical interaction of a tactile probe tip with a surface in order to improve the measurement uncertainty has become very common, but ongoing research is focused on the best modelling of the optical interaction with the optical surface. The development of contactless measurement instruments has several advantages and is attractive due to their non-contact nature and higher measurement speeds than tactile systems [31,32,33,34,35].

Measurements performed on two selected innovative TIMMs were conducted such as to demonstrate improvements in the freeform metrology domain. The aim is to evaluate the measurement results obtained when using different measurement techniques. The first selected material measure represents a high optical quality aspheric surface with nine additional asymmetric steps. The second is an optical quality freeform surface. All the collected data sets obtained using the selected ultra-high precision measuring instruments were evaluated using a robust reference MZ fitting algorithm. Thus, this paper is organised as follows. Section 2 is a description of the selected thermo-invariant material measures and their manufacturing process. In Section 3, a description of the used ultra-high measurement techniques is given. Section 4 details the implemented robust reference MZ fitting algorithms. Finally, Section 5 deals with the evaluation process, main obtained results and analysis.

## 2. Design and Manufacturing of Innovative Thermo-Invariant Material Measures

A number of innovative TIMMs were designed and developed within the European projects IND-FORM and FreeFORM-15SIB01 [36]. Two additional reference TIMMs were recently designed, manufactured and considered for the evaluation of ultra-high precision measurement machines. The first, “TIMM-1”, is designed for the assessment of the mathematical MZ approach with aspheric surfaces. The second, “TIMM-2”, is designed for the evaluation of the MZ approach with industrial freeform surfaces.

The TIMM-1 is an aspheric surface described using the ISO 10110-12 formulation [37] given in Equation (1), where z represents the sag of the surface, r is the radial distance, R is the radius of curvature at the apex of the surface, κ is the conic constant of the conic section and $a_{2m+4}$ are the monomial coefficients. This representation could be used to approximate any symmetric shape with arbitrary accuracy while M is allowed to be large.

(1) $$z\left(r\right)=\frac{r^{2}}{R\left(1+\sqrt{1-\left(1+\kappa \right)\frac{r^{2}}{R^{2}}}\right)}+\overset{M=3}{\underset{m=0}{\sum }}a_{2m+4\;}r^{2m+4}$$

**Figure 3:**
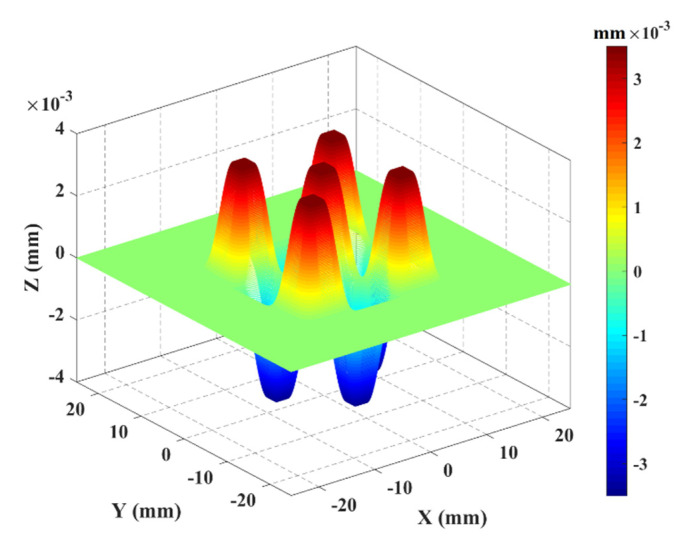
Description of the traceability chain in asphere and freeform metrology.

**Figure 4:**
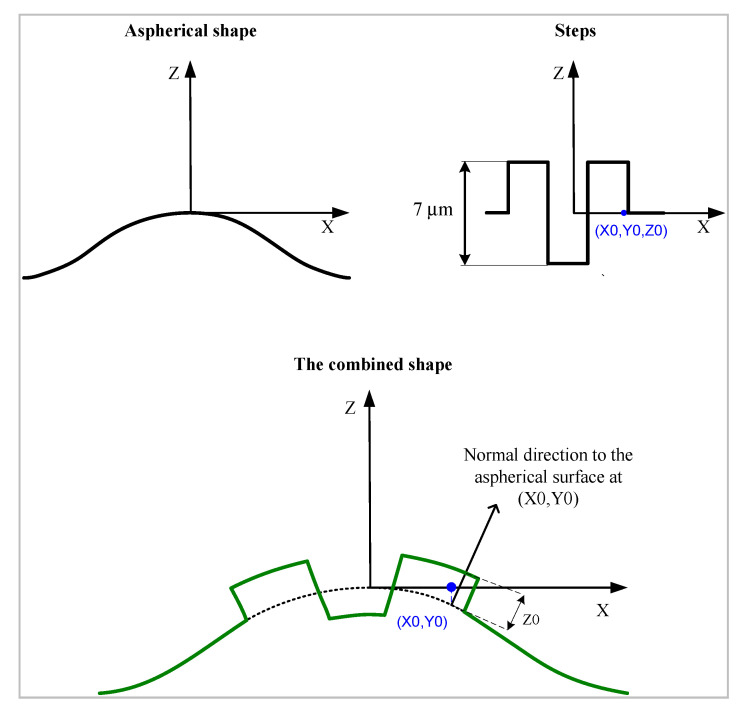
Construction of the thermo-invariant material measure for aspherical geometry TIMM-1 seen in the y = 0 plane. The nominal shape is contained inside two similar aspherical shapes forming the artificial envelope that represent the form error.

Nine steps were added along normal directions to the asphere, as shown in Figure 3. They present an asymmetric distribution along the axis of revolution. The approach for the combination of the steps and the aspheric surface is summarised in Figure 4. These steps represent artificially added form errors (or an artificial envelope) that illustrate the departure from the ideal asphere. They materialise the upper and lower surfaces defining the MZ. In this way, the locations of the significant points defining the MZ are known prior to the process of MZ fitting.

**Figure 5:**
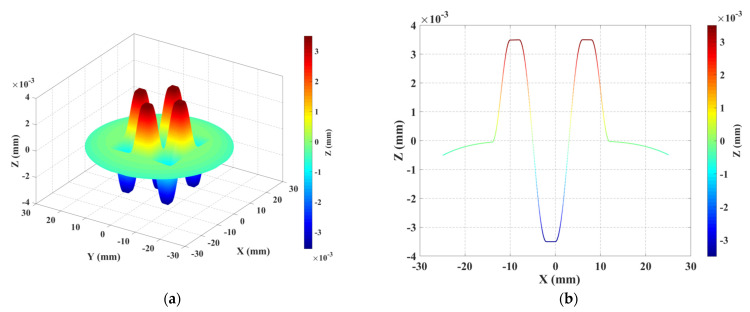
(**a**) Design of the thermo-invariant material measure for aspherical surface (TIMM-1), (**b**) bottom: y = 0 plane. The nominal shape is contained inside two similar aspherical shapes forming the artificial envelope, which represent the form error.

The nominal peak-to-valley (PV) of the steps (defined as the difference between maximum and minimum amplitudes to the nominal shape calculated along the normal direction to the ideal asphere) is equal to 7 µm, which will be used as the nominal MZ value. This value is the smallest amplitude that could be manufactured using the available MRF technology. The final obtained optical surface represents neither axis of symmetry nor degrees of invariance, as illustrated in Figure 5a,b. The nominal shape parameters of the selected asphere are given in Table 1.

**Figure 6:**
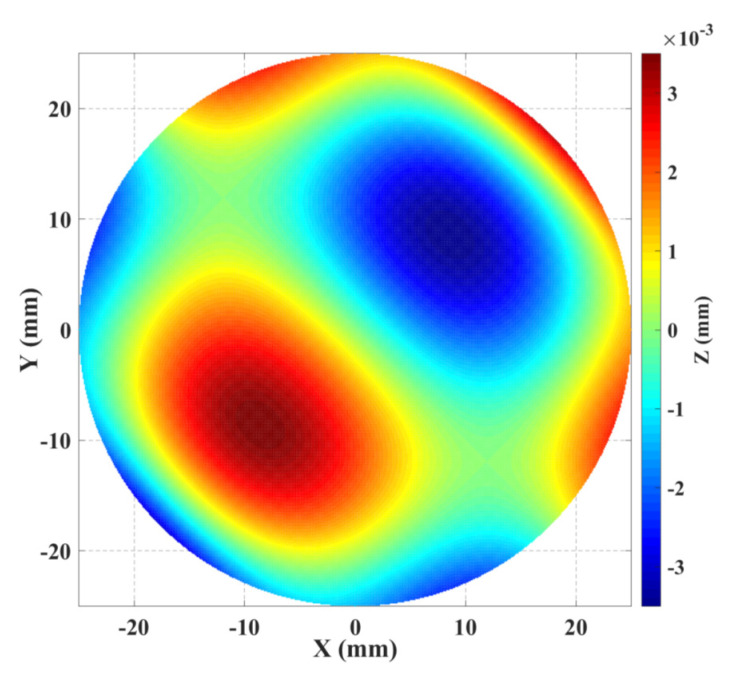
Design of the proposed thermo-invariant material measure for freeform surface TIMM-2 with application in industry.

The proposed TIMM-2 is a freeform surface that has existent applications in industry. It could be incorporated in transparent screens of the oxygen mask embedded in firefighters’ helmets, which allows them to have real time information through augmented reality during action. The shape is described using the explicit polynomial equation presented in (2). The corresponding nominal values of the coefficients $\;{\left\{a_{i}\right\}}_{1\leq i\leq 8}$ were selected according to the constraints imposed by the MRF manufacturing process in terms of amplitude and slope (Table 2) and, as such, the resulting shape has zero degrees of invariance (Figure 6).

(2) $$\begin{array}{ll} Z=a_{1}\left(x^{3}+y^{3}\right) & +a_{2}\left(xy^{2}+x^{2}y\right)+a_{3}\left(x^{5}+y^{5}\right)+a_{4}\left(xy^{4}+x^{4}y\right)\\ & +a_{5}\left(x^{2}y^{3}+x^{3}y^{2}\right)-a_{6}x-a_{7}y-a_{8} \end{array}$$

Both material measures were manufactured using the MRF process. MRF uses a MR polishing fluid with liquid composition that undergoes a change in mechanical properties in the presence of a magnetic field [38]. MR fluid contains very small ferromagnetic particles (0.1 µm) that are organised into chains of particles, forming then a spatial structure resulting in a change in mechanical properties. Without the magnetic field, the particles return progressively to a disorganised state and the initial condition of the overall material is restored. The MR fluid contains four main constituents: water, chemical additives, polishing abrasives (oxide cerium or diamante) and magnetic particles. Nevertheless, water is almost used as a carrier fluid for polishing glasses and silicon substrates without any additional chemical agent.

**Figure 7:**
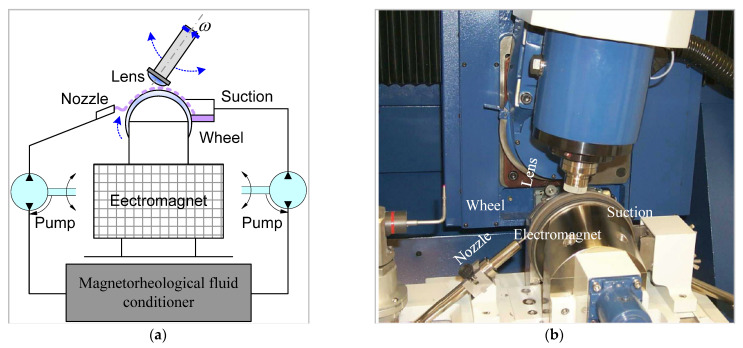
(**a**) Principle of the Q22 multiple-axis computer-controlled magnetorheological finishing (MRF) machine, (**b**) photography of the MRF machine.

Figure 7a,b illustrate the multi-axis computer-controlled MRF machine Q22. The optical element being polished is fixed such that a converging gap can be formed between the element and the rotating spherical wheel. The MR polishing fluid is loaded into the closed-loop fluid delivery system, where fluid properties, such as temperature and viscosity, can be continually monitored. The fluid is driven from the conditioner in a thin ribbon (2 mm × 6 mm) in contact the optical surface, removed by a suction cup and fed back into the conditioner. A local electromagnetic field gradient (0.1 T) is generated by an electromagnet located below the polishing wheel, which causes a change in the mechanical properties of the MR. The MR stiffens in milliseconds and then returns to its original fluid state as it leaves the field, again in milliseconds. The precisely controlled zone of the MR fluid that stiffens becomes the polishing tool. When the optical surface is placed into the fluid, the stiffened fluid ribbon is squeezed from its original thickness, which results in significant shear stress and subsequent polishing pressure over that section of the optical surface [39]. A CNC positioning unit controls the motion of the polishing tool such as to polish the whole workpiece. High-precision surfaces might be achieved by varying the dwell time of the polishing tool on the workpiece surface.

The motion resolution of the used Q22 is equal to 1 μm for linear axes and 2 × 10^−5^ rad for rotational axes. Thus, the MRF process has made it possible to produce classical aspherical glass surfaces with a defect of form (PV) around 0.3 µm and a roughness (Ra) less than 5 nm. The Q22 MRF machine can manufacture aspheres up to 600 mm diameter in the max size.

**Figure 8:**
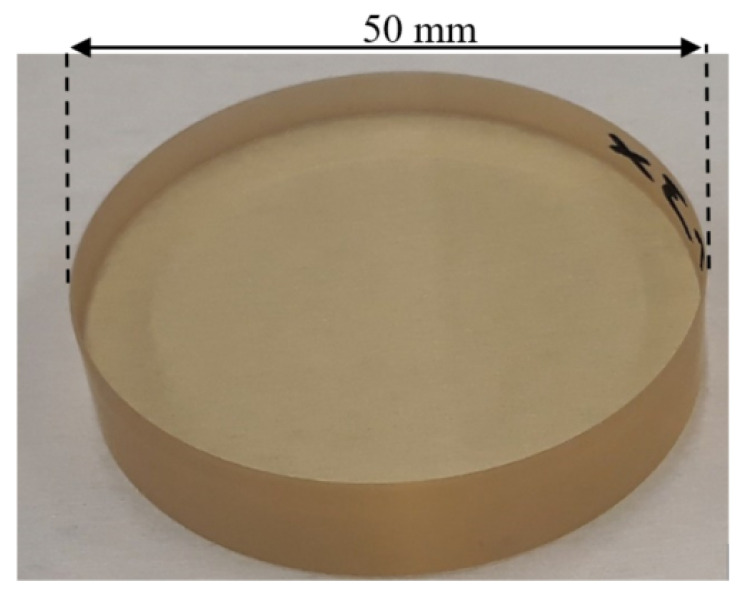
A photograph of the manufactured aspherical artefacts with additional steps slong the normal direction (TIMM-1).

The described accurate MRF process was used to manufacture the two designed innovative material measures TIMM-1 and TIMM-2 made of Zerodur^®^ Class 0 SPECIAL, which is a glass-ceramic with a very low thermal expansion coefficient (less than 0.01 × 10^−6^ K^−1^) [40] (Figure 8). Additional mechanical, optical and chemical properties are given in Table 3.

## 3. Selected Measurement Instruments

**Figure 9:**
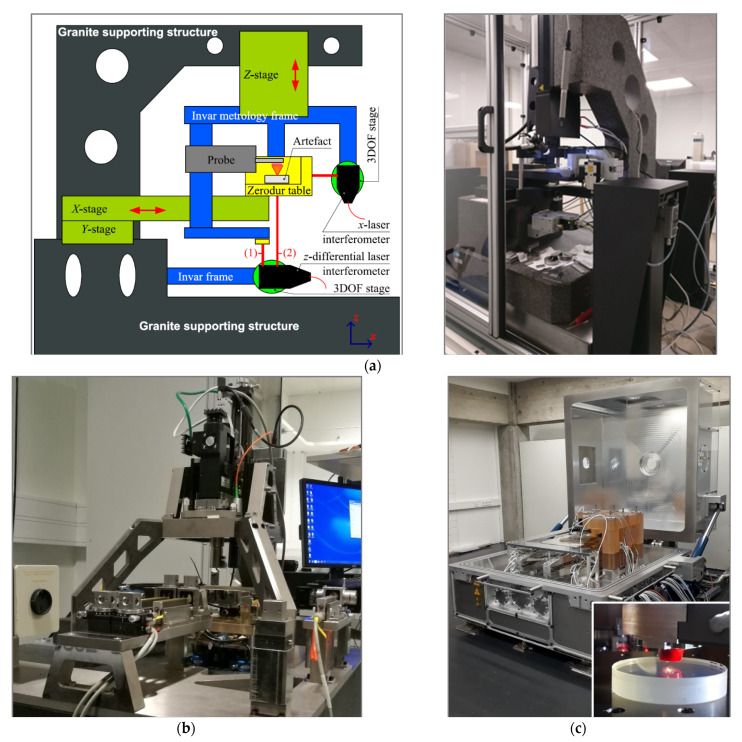
(**a**) LNE’s ultra-high precision primary profilometer, (**b**) VTT’s multi-sensor optical profilometer, (**c**) ITO’s Nano-measuring machine (NPMM-200) and the focus sensor setup. All those measurment instruments are directly traceables to the SI metre definition.

Only ultra-high precision measurement instruments were selected, as given in Table 4. Most of these instruments apply the dissociated metrological structure principle detailed in [23]. Therefore, the selected ultra-high precision instruments are:

- LNE—ultra-high precision primary profilometer: its design has a metrology frame that is separated from the supporting frame [23]. The measured specimen is mounted on a slide way made of Zerodur^®^, which is translated in the horizontal plane, and the motion is tracked in real time by three laser interferometers (Figure 9a), aligned to point at the centre of the contact stylus along the three directions, to minimise Abbe error [41].
- THALES-Agx—Sub-aperture stitching interferometer (SSI) is a Fizeau interferometer with a height range of 6 µm and a multi-axis control system. The lateral measuring range of the SSI is 200 mm with slope angles up to 90° (concave and convex).
- UNOTT—coherence-scanning interferometer (CSI) [42] uses a broadband and spatially extended light source with an interferometric objective to generate low-coherence interference fringes as the instrument scans along the optical axis of the system. The surface topography of a sample is then derived from a combination of the envelope and phase of these interference fringes.
- IPP—LuphoScan 260 HD is a multiple wavelength single point optical probe that performs a spiral scan over the surface and produces high-density 3D data. Scanning is achieved by rotating the object by an air-bearing spindle while the sensor is moved radially and axially using linear stages. A rotary stage keeps the sensor normal to the object surface.
- IPP—MarForm MFU 200 is an optical sensor based on multiple wavelength interferometry. The single point optical probe measures along multiple concentric polar profiles by rotating the spindle and these measuring points are used to generate topography.
- VTT—Multi-sensor optical profilometer is a newly developed instrument based on the measurement of sub-images using coherence scanning interferometer and stitching them together to a high-resolution image (Figure 9b). The horizontal displacements and rotation of the sample between sub-images are tracked using heterodyne laser interferometers. Straight and accurately tracked movements of the sample allow correction of the height difference of the sub-images. The instrument also has a chromatic confocal sensor for fast coarse scans.
- NMIJ—UA3P-4000 is an ultra-high precision profilometer equipped with a single point diamond stylus. The material measures were measured in multiple lines along the *x*-axis of the workpiece coordinate system.
- ITO—Nanopositioning and Nanomeasuring Machine NPMM-200 is equipped with optical focus sensor fixed on a metrological frame made of Zerodur^®^ that holds a number of fiber-coupled laser interferometers to track the relative position of the sample holder (Figure 9c). The single point sensor was used in a null mode; meaning that the machine controlled the z-position of the sample holder such that the sample surface was kept in focus [43].

## 4. Implemented Robust Reference Minimum Zone (MZ) Fitting

The evaluation was based on the determination of the MZ value of each measurement. The MZ value determination problem can be formulated as follows: assume a set of m measured data points ${\left\{p_{i}\right\}}_{1\leq i\leq m}$ and their corresponding orthogonal projections ${\left\{q_{i}\right\}}_{1\leq i\leq m}$ onto a surface described using an implicit equation f(q,s)=0,  where q=(x, y, z) are the coordinates of a given point on the surface and s are the surface’s shape parameters. The MZ fitting problem is formulated as:

(3) $$\underset{x}{min}\phi \left(x\right)\;where\;\underset{1\leq i\leq m}{\phi \left(x\right)=max}f_{i}\left(x\right)$$

where $f_{i}$ denotes the Euclidean distance between the point $p_{i}$ and its corresponding orthogonal projection $q_{i}$. $x\in {\mathbb{R}}^{n}$ can be either the set of intrinsic shape parameters s or the motion parameters m: rotation and translation applied to $\left\{p_{i}\right\}$.

The implemented hybrid trust region method (HTR) was used to solve the optimisation problem in Equation (3) [44]. HTR is an iterative method that involves approximation of the MZ fitting problem using quadratic programming at each iteration and then applying either a trust region step, line search step or curve search step according to the situation at each iteration. This method avoids solving the trust region problem many times.

The uncertainty on the returned MZ value given by HTR algorithm is estimated to be less than 10^−14^ mm. This value was estimated using reference softgauges and does not include the uncertainties resulting from the measurement instruments or measuring process [45].

## 5. Measurements, Results and Discussion

The manufactured two TIMMs were carefully cleaned inside the LNE’s cleanroom before proceeding to measurement. An appropriate cleaning process was investigated and applied in order to eliminate contamination while reducing measurement uncertainty. In fact, the presence of these undesirable substances on the surface causes the obtained MZ value to heavily deviate from the actual one (in absence of the particles). The adopted iterative cleaning process consists of the following steps: (1) Triton and Foam, (2) Ultrasonic bath Acetone during 10 min, (3) Ethanol ultrasonic bath during 10 min, (4) Rinse with milliQ water, (5) Compressed air dry and (6) Control of the surface using an accurate optical microscope.

Within the fixed rule in the procedure, each material measure is probed/scanned three times by the same ultra-high precision measurement system under restrictive environment condition. In particular, all measurements were performed inside a metrology cleanroom where the temperature is controlled to 20 °C. The handling of the material measures is carefully done.

Once the measurement datasets were collected, manual removal approach of outliers was conducted since there are no automatic processes and studies that can be applied for such data. Afterwards, the MZ values were extracted using the implemented robust reference HTR algorithm and the expanded standard uncertainties were estimated when applying the type-A evaluation, according to the GUM [45].

**Figure 10:**
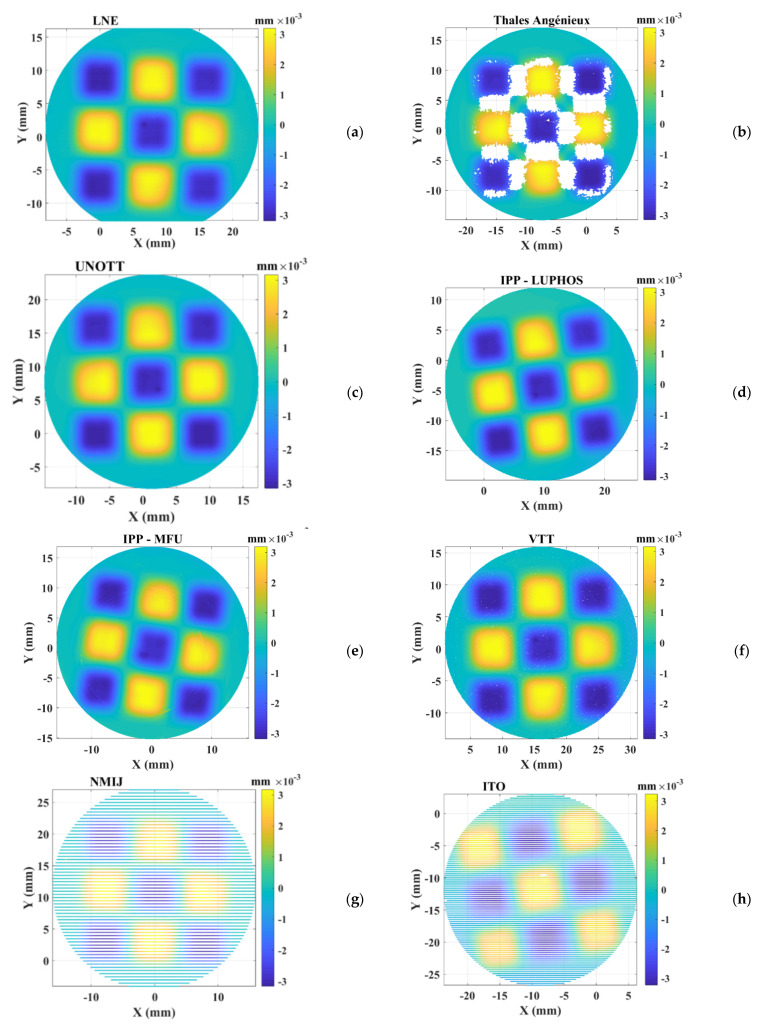
Illustration of the obtained residuals when applying the implemented robust reference minmax fitting algorithm on measured datasets (TIMM-1) using the described ultra-high precision measurement machines at LNE (**a**), Thales-Agx (**b**), UNOTT (**c**), IPP-LUPHOS (**d**), IPP-MFU (**e**), VTT(**f**), NMIJ (**g**) and ITO (**h**).

The obtained residual maps of the measurements are illustrated in Figure 10. Thus, measurements made by IPP and ITO seem to be slightly rotated around the *z*-axis compared to the other measurements. This is due to the initial positioning of the TIMM-1 before proceeding to the measurement. However, this has no effect on the final value of MZ since the nominal shape is rotationally symmetric. The residuals calculated on measurement datasets made by Thales-Agx when using the sub-aperture stitching interferometer show some missing areas especially due to measurement system limitations on 3D surfaces with high slope. This has no effect of the final MZ value since the regions defining the minimum zone were completely detected. Otherwise, most selected ultra-high precision measurement instruments return measured datasets covering the whole surfaces of the TIMMs.

**Figure 11:**
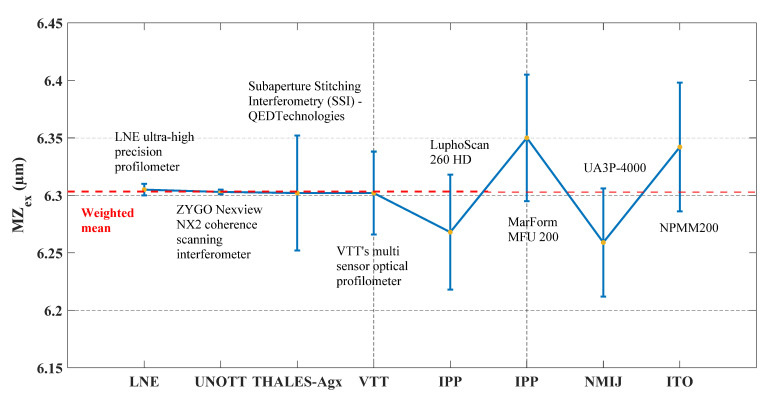
Illustration of the obtained MZ_ex_ values obtained by applying the implemented robust reference minmax fitting algorithm (hybrid trust region) and the estimated expanded standard uncertainties for TIMM-1. The obtained MZ_ex_ values could be compared to the weighted mean value.

**Figure 12:**
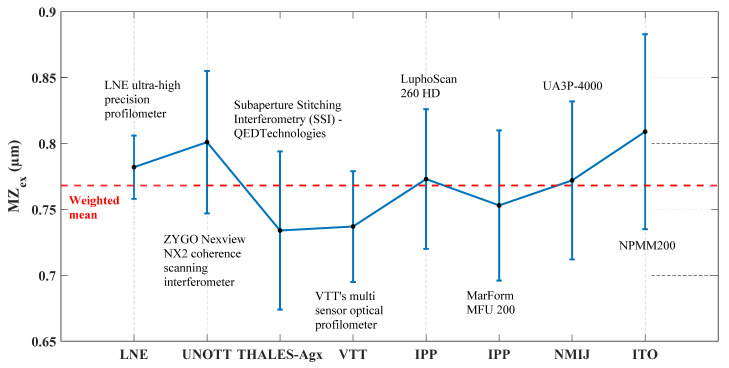
Illustration of the obtained MZ_ex_ values obtained by applying the implemented robust reference minmax fitting algorithm (Hybrid Trust Region) and the estimated expanded standard uncertainties for TIMM-2.

Furthermore, the obtained experimental values of MZ (denoted *MZ_ex_*) for both material measures (TIMM-1 and TIMM-2) are extracted and presented in Figure 11 and Figure 12. The Key Comparison Reference Value *MZ_ref_* ([45]) is calculated using the weighted mean given in Equation (4):

(4) $$MZ_{ref}=\;\underset{i}{\sum }\omega_{i}.MZ_{ex,i}$$

where

(5) $$\omega_{i}=C\frac{1}{\left[u{\left(MZ_{ex,i}\right)}^{2}\right]}$$

and

(6) $$C=\;\frac{1}{\sum_{i}\frac{1}{\left[u{\left(MZ_{ex,i}\right)}^{2}\right]}}$$

The uncertainty of the weighted mean is calculated using the Equation (7). A coverage factor *k* = 2 is used for the calculation of the expanded standard uncertainty.

(7) $$u\left(MZ_{ref}\right)=\;\sqrt{\frac{1}{\sum_{i}\frac{1}{\left[u{\left(MZ_{ex,i}\right)}^{2}\right]}}}=\sqrt{C}$$

Figure 11 illustrates the average experimental *MZ_ex_* values with the respective expanded standard uncertainty for TIMM-1. The calculated Key Comparison Reference Value *MZ_ref_* is equal to 6.303 µm with an associated expanded standard uncertainty of 2 nm. Based on this value of *MZ_ref_*, a deviation from the theoretical value of MZ (*MZ_th_* = 7 m) by 697 nm can be seen. The deviation (*MZ_th_*—*MZ_ref_*) is due to the manufacturing MRF process. Thus, a small error in the estimation of the wear rate of the tool used in the MR process may lead to significant form errors. The manufacturing of TIMM-1, because of its complex shape compared to a classical asphere, took approximately nine hours while a normal MRF cycle takes fifteen to forty-five minutes, which may explain this deviation.

The probability density functions based on kernel density estimation were calculated for all collected measurement data. The obtained functions present similar Gaussian shape which could validate the measurements qualities.

The average *MZ_ex_* and expanded standard uncertainties obtained from measurements are respectively equal to 6.305 µm and 5 nm for LNE, and 6.303 µm and 2 nm for UNOTT. These two measurements could be considered as the most accurate. In addition, the average *MZ_ex_* value obtained from Thales-Agx measurements is too close to the LNE and UNOTT values.

Nevertheless, measurements on TIMM-1 made by all participants present a good agreement as shown in Figure 11, even if deviations can be observed (with comparison to the *MZ_ex_* values given by LNE, UNOTT and Thales-Agx) due to systematic and random errors that could be compensated.

Moreover, the obtained results prove the capabilities of all participants to carry out measurements on aspherical surface with high accuracy. Once the TIMM-1 is calibrated by any ultra-high precision measuring machine with a low uncertainty, the collected measured data could be used for testing or verifying implemented minimax industrial algorithms. As consequence, the MZ value returned by the minimax industrial algorithms will has a guaranteed traceability to the SI unit meter definition.

Unlike TIMM-1, the obtained *MZ_ex_* values for TIMM-2 are more dispersed. The obtained *MZ_ex_* values are plotted in Figure 12. The Key Comparison Reference Value *MZ_ref_* is equal to 0.768 µm with an associated expanded standard uncertainty of 0.016 µm. The measurement made by LNE has the lowest expanded standard uncertainty (0.024 µm).

The obtained results show the interest of the design of the proposed TIMMs for MZ fitting. Both optical and tactile measuring systems provide results with good agreement. Furthermore, the collected results demonstrate the establishment of a traceable reference full metrology chain including ultra-high precision measurement instruments, innovative thermo-invariant material measures and robust reference minimax fitting algorithms accurate at few tens of nanometers for aspherical and freeform optical surfaces. However, the obtained values are still sensitive to the presence of outliers among the collected data. Hence, clear pre-processing steps for filtering and outlier removal must be defined.

## 6. Conclusions

This paper presents the capability to use several ultra-high precision measurement machines for the evaluation of innovative optical aspheric and freeform surfaces. The evaluation was made based on the obtained form errors estimated with the implemented robust reference HTR algorithm for minimum zone fitting. This procedure includes the main three components of the traceable reference metrology chain of aspheric and freeform surfaces: (1) ultra-high precision measuring machines, (2) thermo-invariant material measures and (3) reference algorithms.

Measurements were made on two developed TIMMs: TIMM-1 and TIMM-2. TIMM-1 is designed for the assessment of MZ fitting of aspherical surfaces while TIMM-2 is designed for freeform surfaces with applications in industry.

A number of ultra-high precision measurement machines were selected for the evaluation tests. The overall results show that there is a difference between the theoretical and measured form error of TIMM-1 and TIMM-2, which is likely due to the MRF manufacturing process. MRF process usually requests less than an hour to manufacture classical optical aspheres, while 9 h was taken for the manufacturing of the designed TIMMs, which caused the wear to appear in the tool, and then the deviation between both theoretical and manufactured TIMMs.

Furthermore, good agreement between the obtained results was observed; in particular, results obtained with the LNE ultra high precision primary profilometer and the UNOTT improved coherence-scanning interferometer. The expanded standard uncertainties on the weighted mean value of the *MZ_ex_* for the two material measures did not exceed 16 nm. In addition, it is to be noted that these uncertainties were achieved in the case where the theoretical amplitudes are small (less than 7 µm).

The perspectives of this work are:

- similar measurements tests might be conducted on material measures with higher amplitudes;
- clear pre-processing approaches for filtering and outlier removal must be established since the obtained results are highly sensitive to each point in the data set;
- the implemented MZ fitting algorithm (HTR) considers motion parameters only. Indeed the determination of shape parameters might prove important and should be studied;
- investigation of more robust fitting criteria than MZ. In fact, MZ criterion could be used with caution since it is highly affected by outliers and no standard outlier removal method exists;
- development of reference softgauges with a non-vertex solution in the case of freeform surfaces;
- investigation of a reference metrology accurate at the nanometre level for waviness and areal texture of aspherical and freeform surfaces. This metrology could include the development of new material measures, improved ultra-high measurement instruments as well as references algorithms and softgauges. Filtering algorithms could be also studied.

## Figures and Tables

**Table 1:** Nominal shape parameters for TIMM-1.

Parameter	Value
R (mm)	9.127 × 10^40^
κ	−1
$a_{4}$ $\;({\mathrm{mm}}^{-3})$	1.278 × 10^−9^
$a_{6}$ $\;({\mathrm{mm}}^{-5})$	7.922 × 10^−16^
$a_{8}$ $\;({\mathrm{mm}}^{-7})$	−1.859 × 10^−18^
$a_{10}$ $\;({\mathrm{mm}}^{-9})$	1.733 × 10^−21^

**Table 2:** Nominal shape parameters for TIMM-2.

Parameter	Value
$a_{1}\;\left({\mathrm{mm}}^{-2}\right)$	9.792 × 10^−7^
$a_{2}\;\left({\mathrm{mm}}^{-2}\right)$	4.940 × 10^−7^
$a_{3}\;\left({\mathrm{mm}}^{-4}\right)$	−6.31 × 10^−10^
$a_{4}\;\left({\mathrm{mm}}^{-4}\right)$	−3.086 × 10^−10^
$a_{5}\left({\mathrm{mm}}^{-4}\right)$	2.551 × 10^−10^
$a_{6}$	3.087 × 10^−4^
$a_{7}\;$	3.087 × 10^−4^
$a_{8}\left(\mathrm{mm}\right)$	−6.876 × 10^−10^

**Table 3:** Typical mechanical, optical and chemical properties of Zerodur^®^ [40].

Parameter	Value
Young‘s modulus E at 20 °C [GPa]-mean value	90.3
Knoop Hardness HK 0,1/20 (ISO9385)	620
Density [g/cm^3^]	2.53
Refractive index *n_d_*	1.5424
Stress optical coefficient *K* at *λ* = 589.3 nm [10^−6^ MPa^−1^]	3.0
Acid resistance class (ISO 8424)	1.0

**Table 4:** Description of the measurement range and resolution of the selected ultra-high precision measurement systems, and number of collected data points.

Ultra-High Precion Measurment Machines	Measurment Range X, Y and Z (in mm)	Resolution (in nm)	Number of Recorded Data Points	
			TIMM-1	TIMM-2
LNE—ultra-high precision primary profilometer	100 × 100 × 50	0.09	247,590	187,453
THALES-Agx—Sub-aperture Stitching Interferometer (SSI)	200 × 200 × 6	0.4	192,771	94,486
UNOTT—Coherence-Scanning Interferometer (CSI)	100 × 100 × 20	0.12	360,291	263,224
IPP—LuphoScan 260 HD	400 × 400 × 100	1	90,646	90,646
IPP—MarForm MFU 200	180 × 180 × 100	0.5	321,657	321,657
VTT—Multi-sensor optical profilometer		0.15	260,830	465,849
NMIJ—UA3P-4000	100 × 100 × 35	0.3	160,504	117,313
ITO—Nanopositioning and Nanomeasuring Machine NPMM-200	200 × 200 × 25	0.02	237,151	234,739

## Data Availability

Data sharing is not applicable to this article.
